# Epigenetic Mechanisms Underlying the Dynamic Expression of Cancer-Testis Genes, *PAGE2*, *-2B* and *SPANX-B*, during Mesenchymal-to-Epithelial Transition

**DOI:** 10.1371/journal.pone.0107905

**Published:** 2014-09-17

**Authors:** Sinem Yilmaz-Ozcan, Asli Sade, Baris Kucukkaraduman, Yasemin Kaygusuz, Kerem Mert Senses, Sreeparna Banerjee, Ali Osmay Gure

**Affiliations:** 1 Department of Molecular Biology and Genetics, Bilkent University, Ankara, Turkey; 2 Department of Biological Sciences, Middle East Technical University, Ankara, Turkey; The University of Texas MD Anderson Cancer Center, United States of America

## Abstract

Cancer-testis (CT) genes are expressed in various cancers but not in normal tissues other than in cells of the germline. Although DNA demethylation of promoter-proximal CpGs of CT genes is linked to their expression in cancer, the mechanisms leading to demethylation are unknown. To elucidate such mechanisms we chose to study the Caco-2 colorectal cancer cell line during the course of its spontaneous differentiation *in vitro*, as we found CT genes, in particular *PAGE2, -2B* and *SPANX-B*, to be up-regulated during this process. Differentiation of these cells resulted in a mesenchymal-to-epithelial transition (MET) as evidenced by the gain of epithelial markers CDX2, Claudin-4 and E-cadherin, and a concomitant loss of mesenchymal markers Vimentin, Fibronectin-1 and Transgelin. PAGE2 and SPAN-X up-regulation was accompanied by an increase in Ten-eleven translocation-2 (TET2) expression and cytosine 5-hydroxymethylation as well as the disassociation of heterochromatin protein 1 and the polycomb repressive complex 2 protein EZH2 from promoter-proximal regions of these genes. Reversal of differentiation resulted in down-regulation of *PAGE2, -2B* and *SPANX-B*, and induction of epithelial-to-mesenchymal transition (EMT) markers, demonstrating the dynamic nature of CT gene regulation in this model.

## Introduction

Cancer-testis (CT) or cancer-germline genes are expressed in tumors originating from various tissues, as well as in normal germline and trophoblast cells, but are generally silent in other normal tissues of the adult [1]–[3]. More than 100 different CT genes can be grouped according to homology into families [2]. Despite the lack of sequence similarity between CT genes from different families, re-expression of all CT genes in tumors has been associated with demethylation of CpG residues within their promoter-proximal regions [4]. This shared mechanism of expression regulation is most likely the reason for their coordinate expression in cancer [5]–[7]. However, the exact mechanism by which DNA demethylation occurs at CT gene promoter-proximal regions in cancers is unknown. CT genes show mostly a heterogeneous expression pattern in tumors [8]–[10]; in contrast to their expression in testis, which is demarcated and orderly [11]. A study in which stem-like and non-stem like cells of breast cancer were selectively killed, revealed that CT gene expression is generally a feature of more differentiated, non-stem cells [12]. Similarly, in melanoma, a subgroup of cells with more epithelial features express CT genes, when cells with mesenchymal features don't [13]. Interestingly, melanoma cells can switch between these two classes *in vivo*, suggesting that tumor heterogeneity, as defined by CT gene expression, might represent a transitional phase similar to the switch between epithelial and mesenchymal phenotypes. Indeed, mesenchymal-to-epithelial transition (MET) is associated with the induction of CT gene expression [14]. To define mechanisms involved in regulating CT gene expression in cancer and during MET, we chose to study the Caco-2 spontaneous differentiation model which demonstrates features of MET and EMT during differentiation and de-differentiation, respectively. Our data reveal that the dynamic regulation of the two CT genes, *PAGE* and *SPANX-B* in this model system, involves alterations of polycomb repressive complex 2 (PRC2) and heterochromatin protein 1 (HP1) occupancy within their promoter-proximal regions, with concordant changes in TET expression and cytosine hydroxymethylation (hmC) levels.

## Methods

### Cell lines, induction of differentiation and de-differentiation

The Caco-2 cell line was obtained from the SAP Enstitusu (Ankara, Turkey). HCT116 (colorectal) and Mahlavu (hepatocellular) cancer cell lines were obtained from LGC Standards, Middlesex, UK. A lung cancer cell line (SK-LC-17) was from the Memorial Sloan Kettering Cancer Center, NY, USA. Caco-2 cells were grown in EMEM and others in RPMI, supplemented with 20% FBS, 2 mM L-glutamine, 0.1 mM non-essential amino acids, 1.5 g/L sodium bicarbonate and 1 mM sodium pyruvate. All cell culture media and supplements were purchased from Biochrom AG, Berlin, Germany. The first day cells reached confluence was designated day 0. Cells grown in parallel cultures were used to determine phenotypic changes at days 0, 5, 10, 20 and 30, post-confluence. Additional measures of differentiation for cells used in this study have been reported elsewhere [15]. To induce dedifferentiation, cells at the 20th day of differentiation were detached and replated at about 50% confluence and RNA and protein were harvested 5 days following replating.

### *In silico* analysis of CT and EMT gene expression

Expression data contained within GSE1614 [16] was GC-RMA normalized using GeneSpring v. 11.0. CT gene expression was analyzed based on 31 probesets in corresponding to 23 CT genes from 7 families (**Figure S2**). An interpretation was generated with an entity list composed of EMT related genes as defined by Loboda et al. [17], at three different time points. Genes for validation were selected among those for which significant differences of expression (p<0.05) was observed by one way ANOVA test and Bonferroni FWER correction, when proliferating cells were compared to those at day 15.

### Quantitative RT-PCR

Total RNA was isolated using the Trizol reagent (Ambion, Foster City, CA, USA) and treated with DNAse I (Ambion, Foster City, CA, USA). 200 ng of RNA was reverse transcribed using Revert-Aid first strand cDNA synthesis kit (Thermo Fisher Scientific, Boston, MA, USA). PCR reactions were performed using an ABI 7500 thermal cycler (Applied Biosystems, Carlsbad, CA, USA). All reactions were performed according to manufacturer's recommendations. TaqMan Gene Expression Assays (Applied Biosystems, Carlsbad, CA, USA) were used for the following: GAPDH (4352934E), SPANX-B (Hs02387419_gH), PAGE2 and -2B (Hs03805505_mH), GAGE (Hs00275620_m1), SSX4 (Hs023441531_m1), NY-ESO-1 (Hs00265824_m1), and MAGE-A3 (Hs00366532_m1). SYBR Green master mix with ROX reference dye (Applied Biosystems, Carlsbad, CA, USA) was used to determine *CDH1, CDX2, CLDN4, VIM, FN1* and *TAGLN* expression (**Table S1**). Cycling conditions for these assays were 50°C for 2 min., 95°C for 10 min., followed by 40 cycles of 94°C for 15 sec., 60–65°C for 1 min. Relative expression was calculated by the ΔΔCt method [18]. All samples were analyzed in triplicates and all experiments were repeated at least twice.

### Promoter methylation analysis

Genomic DNA was isolated by Proteinase K treatment, following a phenol-chloroform extraction protocol. Bisulphite treatment of 200 ng genomic DNA was performed using Zymo DNA Methylation Gold Kit (Zymo Research, Irvine, CA, USA). Bisulphite modified DNA was stored at −20°C and used for PCR within 2 months. Two rounds of DNA amplification were performed using One Taq Hot Start DNA polymerase (New England Bioscience/NEB, Ipswich, MA, USA) using a Perkin Elmer 9700 thermal cycler (Applied Biosystems, Carlsbad, CA, USA). Primers used are given in **Table S1**. PCR products were gel extracted using the QIAGEN gel extraction kit (Qiagen, Hilden, Germany) and cloned into pCR2.1 (Invitrogen, Carlsbad, CA, USA). Plasmid DNA was purified using the QIAprep Spin Miniprep Kit (Qiagen, Hilden, Germany) from at least ten clones, and sequence analyzed by IONTEK (Istanbul, Turkey).

### 5-hydroxymethyl cytosine analysis

Caco-2 genomic DNA (gDNA) was sheared by probe sonication (30 sec. on, 30 sec. off, 5 cycles) to obtain 200–600 bp. fragments assessed by 1% agarose gel electrophoretic analysis. Immunoprecipitation was performed using the hMEDIP kit (Abcam, Cambridge, UK) according to manufacturer's instructions. 5 pg of control DNA was spiked into 500 ng of gDNA to use as an internal control. Positive and negative controls of the kit were included in all experiments. 2 µl from the eluted DNA was used as template for quantitative RT-PCR using 2 X SYBR Green master mix with ROX reference dye (Applied Biosystems, Carlsbad, CA, USA) with the primers given in **Tables S1 and S2**. Primer efficiencies were controlled. Cycling conditions were 50°C for 2 min., 95°C for 10 min. followed by 40 cycles of 94°C for 15 sec., 60°C for 1 min. Shared genomic DNA was included in quantitative RT-PCR to calculate % input.

### Immunofluorescence microscopy

Cells attached to glass slides by centrifugation using the Shandon CytoSpin3 (Thermo Scientific, Waltham, MA, USA) were immediately fixed in 2% formaldehyde/PBS at room temperature for 15 min. Fixed cells were permeabilized in 0.2% Triton X-PBS for 10 min. followed by blocking with 1% BSA in 0.1% PBS-Tween for 1 hour. Incubations with the primary antibody, diluted at 1∶50, were performed overnight at 4°C. Secondary antibody was added at 1∶200, following washing in 0.1% PBS-Tween for 5 min. for 3 times, and incubated with cells for 45 min. at room temperature (see **Table S3** for the complete antibody list). Stained samples were mounted with mounting medium (Santa Cruz Biotechnology, Santa Cruz, CA, USA) containing DAPI solution. Cell lines used as positive controls were Mahlavu (PAGE-2,-2B and SPANXB), MDA-MB 231 (VIM), MCF-7 (TAGLN) and SW620 (CDX2, FN1). Negative controls were combinations of primary antibodies with un-related secondary antibodies. All images were obtained using an AxioCam MRc5 image capture device (Carl Zeiss, Oberkochen, Germany).

### Western analysis

Cell lysates (extracted with RIPA buffer) separated on 4–12% Novex Bis-Tris SDS gels (Invitrogen, Carlsbad, CA, USA) were transferred to Immobilon-PSQ membranes (Millipore Corp. Bedford, MA, USA) with an Invitrogen western blotting system (Invitrogen. Carlsbad, CA, USA). Following blocking with 5% milk powder in 0.02% PBS-T, blots were incubated with primary antibody overnight at 4°C. Primary antibody dilutions were 1∶1000 for CDX2, fibronectin, vimentin and transgelin, 1∶2500 for β-actin and 1∶100 for SPANX-B and PAGE-2,-2B antibodies. HRP conjugated secondary antibody (Abcam, Cambridge, UK) was used at a 1∶5000 dilution and incubated at room temperature for 1 hour. Signals were detected using the ECL luminescence assay (BioRad, Hercules, CA, USA).

### Chromatin Immunoprecipitation

Chromatin Immunoprecipitation (ChIP) was performed as previously described [15]. Briefly, formaldehyde cross-linked cell constituents were precipitated by proten A sepharose beads coupled to antibodies against EZH2, HP-1 or H3K27m3 (Abcam, Cambridge, UK), as well as isotype-specific control. Precipitated DNA was amplified using primers specific for *PAGE2*, *-2* or *SPANX-B* promoter sequences (**Tables S1 and S2**), following de-crosslinking.

## Results

### CT gene expression during Caco-2 spontaneous differentiation (Caco-2 SD)

**Figure 1:**
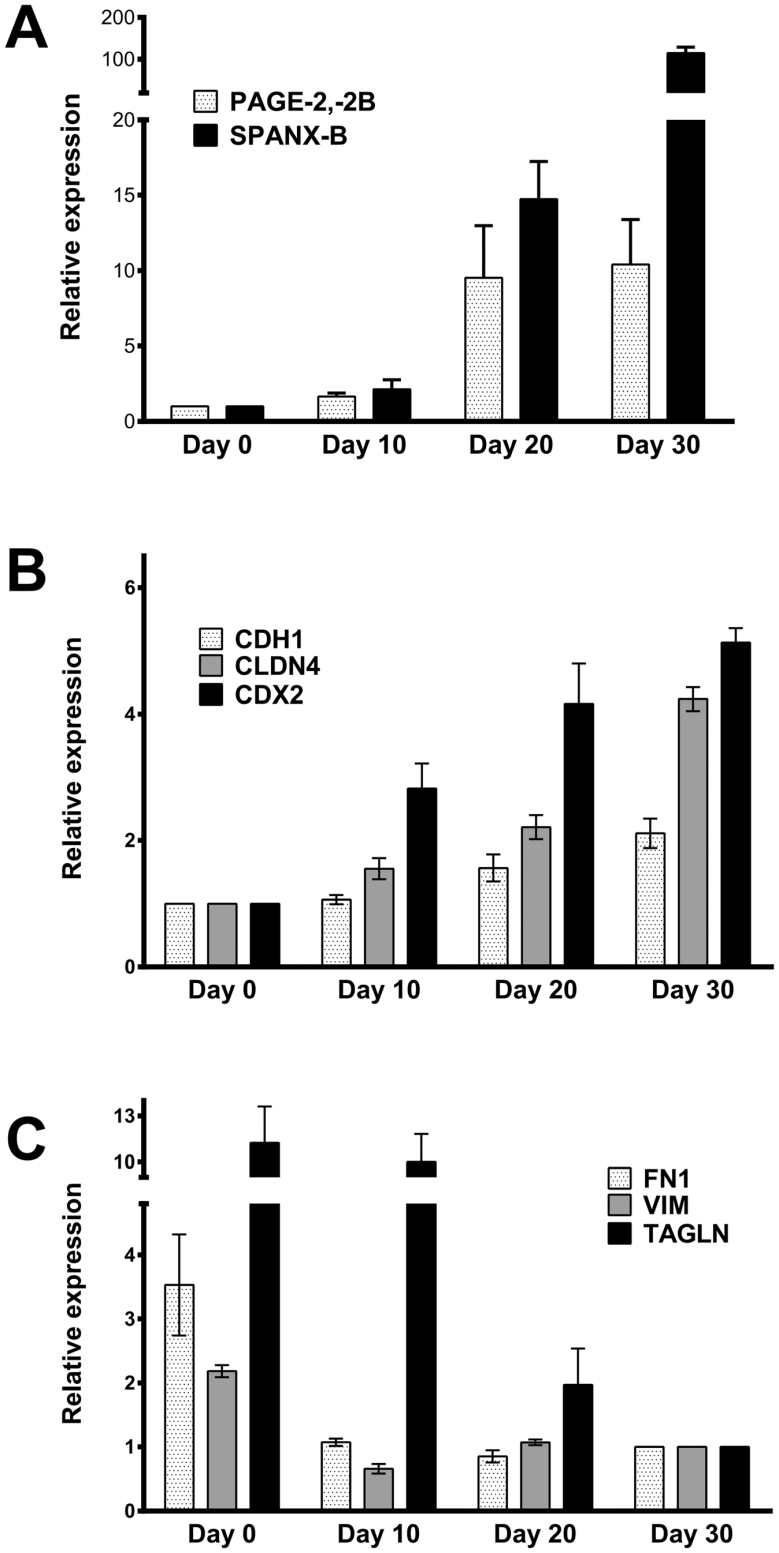
Up-regulation of CT genes in parallel to MET in the Caco-2 SD model. Relative mRNA expression of CT genes (*PAGE2*, *-2B* and *SPANXB*) (**A**), epithelial genes (*E-cadherin (CDH1)*, *claudin 4(CLDN4)*, *CDX2*) (**B**), and mesenchymal genes (*fibronectin 1 (FN1), vimentin (VIM)*, *transgelin (TAGLN)*) (**C**) as determined by quantitative PCR at days 0, 10, 20 and 30 post-confluence. Data represent average of two experiments. Change in expression levels for all genes between days 0 and 30 is statistically significant (P<0.0001, by two way ANOVA with Tukey's post hoc test).

The undifferentiated colorectal cancer cell line Caco-2 undergoes enterocytic differentiation upon reaching confluence *in vitro* [19], [20]. Gradual differentiation has been observed up to 30 days post-confluence as evidenced by the up-regulation of various differentiation-associated genes including sucrase-isomaltase, alkaline phosphatase and carcinoembryogenic antigen (CEA), (**Figure S1**) [15]. An *in silico* analysis of CT gene expression as defined by 31 probesets in the GSE1614 dataset, which contains gene expression data for the Caco-2 SD model obtained during differentiation (proliferating (2^nd^ day), post-proliferation-undifferentiated (8^th^ day), and post-proliferation-differentiated (15^th^ day)) [16], revealed modest up-regulation of almost all CT genes during differentiation (**Figure S2**). We chose to validate the change in expression of six CT gene/gene families by quantitative RT-PCR in differentiating Caco-2 cells *in vitro*. *GAGE, MAGE-A3, NY-ESO-1* and *SSX4* transcripts were undetectable on the first day of confluence, as well as at later time points (data not shown). However, significant up-regulation of *PAGE2* (*2 and 2B*), and *SPANX-B* genes was evident (**Figure 1**).

### *PAGE2* and *SPANX-B* expression follow MET in the Caco-2 SD model

Spontaneous differentiation of Caco-2 *in vitro* has been reported to result in MET [21], [22]. To determine if this occurred in parallel to the up-regulation of *PAGE2* and *SPANX-B*, we analyzed the GSE1614 dataset for the expression of genes representing EMT in colorectal cancer [17], and selected 6 genes to be validated in our model. Analysis of mRNA and protein expression of these revealed a decrease in mesenchymal genes (*vimentin, fibronectin 1* and *transgelin*) with a concomitant increase in expression of epithelial genes (*CDX2, claudin-4* and *E-cadherin*) as the cells differentiated, demonstrating that the increase in CT gene expression occurs simultaneously with MET in this model (**Figure 1** **& Figure S3**).

### PAGE2, SPANX-B and EMT gene expression *in situ*

**Figure 2:**
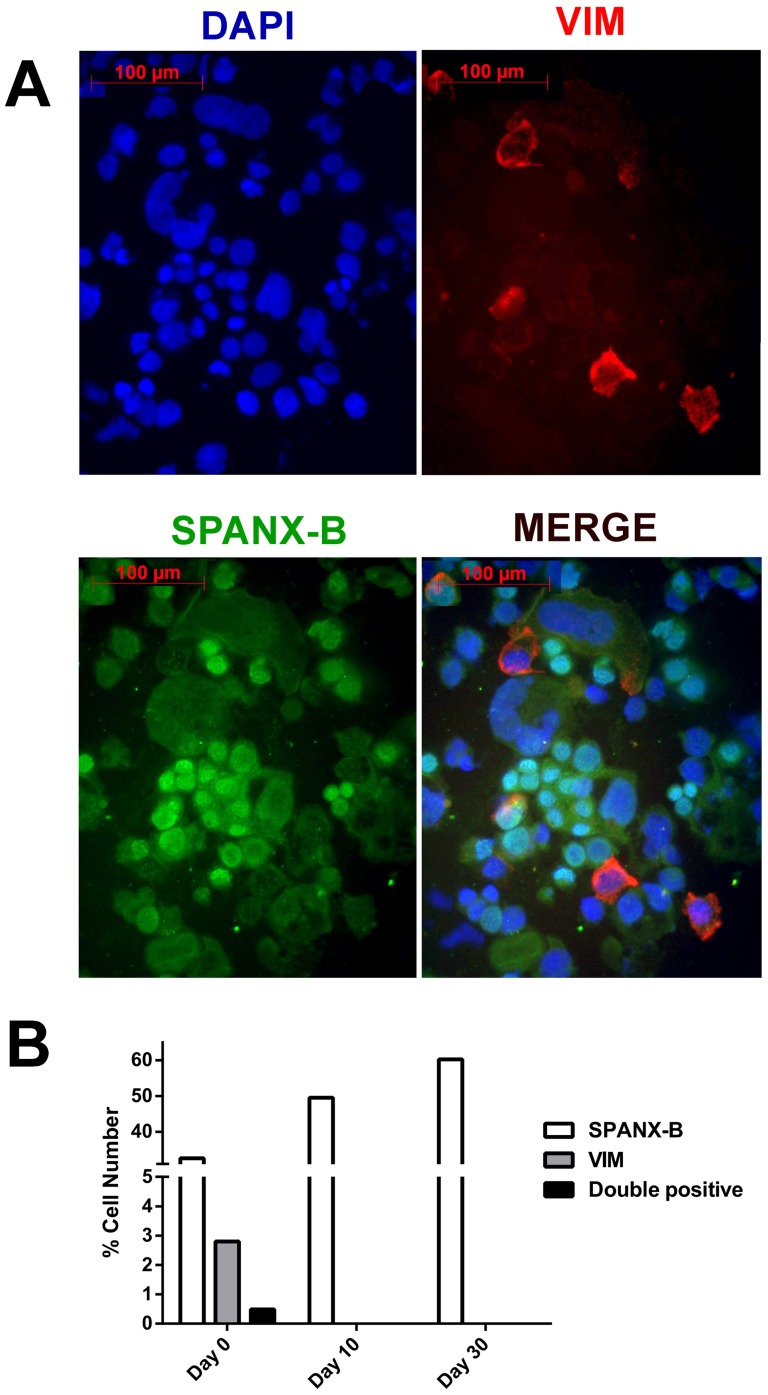
SPANX-B and vimentin expression are mutually exclusive in differentiating Caco-2 cells. Immunofluorescent staining of differentiating Caco-2 cells with DAPI counterstaining reveals a gradual increase in nuclear SPANX-B (green) with a concomitant decrease in cytoplasmic vimentin expression (red); (20× magnification) (**A**). Less than 1% of SPANX-B positive cells showed staining for vimentin on day 0 (**B**).

To study if the changes in protein expression of CT and EMT genes occurred simultaneously in the same cells, we performed double immunofluorescence staining during differentiation. A gradual loss of mesenchymal markers was observed as cells differentiated, with a concomitant increase in epithelial genes and CT genes. SPANX-B and PAGE-2 were frequently co-expressed with the epithelial marker CDX2 in the same cells but almost never with VIM or FN1 (**Figures 2** **,** **3** and **Figures S4, S5, S6, S7**).

**Figure 3:**
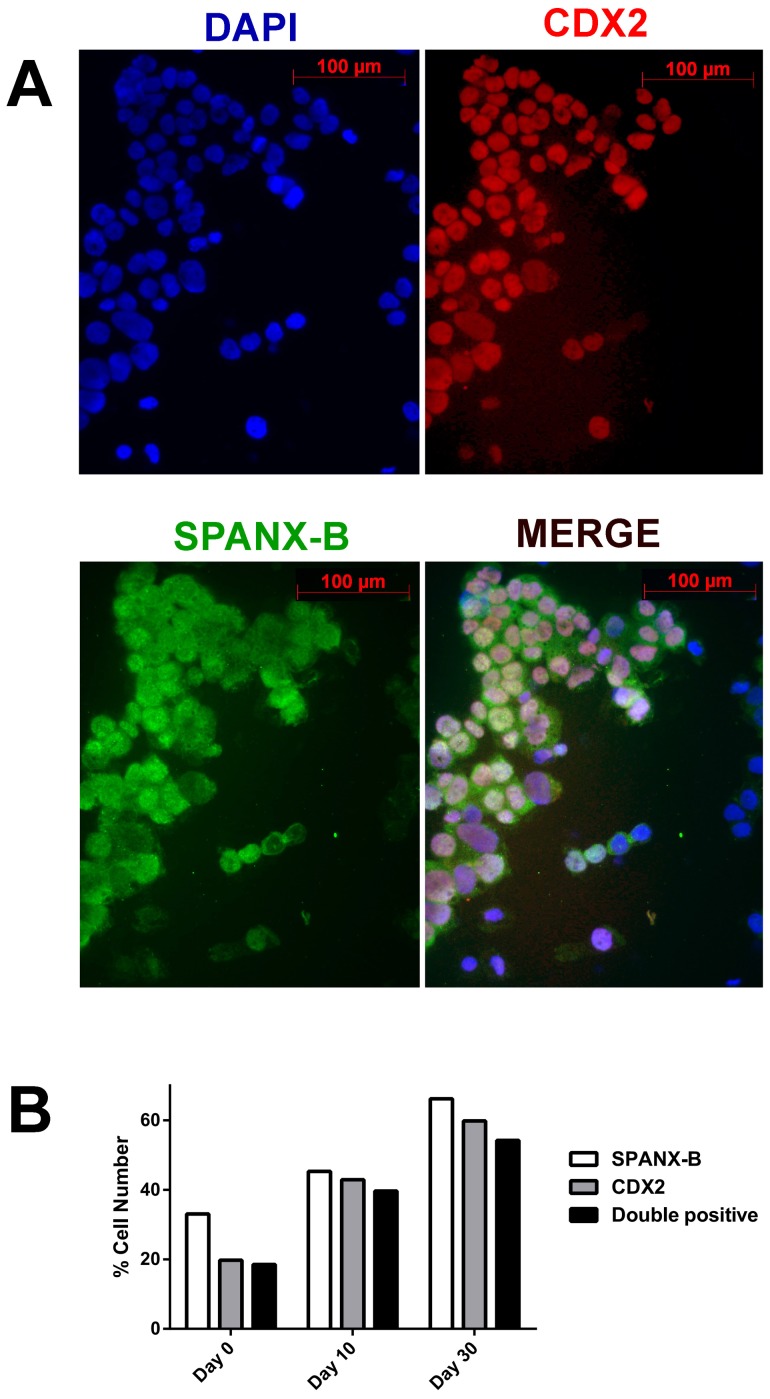
Nuclear co-localization of CDX2 and SPANX-B in differentiating Caco-2 cells. Immunofluorescent staining of differentiating Caco-2 cells with DAPI counterstaining reveals overlapping SPANX-B (Alexa Fluor 488: green) and CDX2 (Alexa Fluor 568: red) expression; (20× magnification) (**A**). More than 60 to 80% of the cells show double-labeling when analyzed quantitatively (**B**).

### PAGE2 and SPANX-B expression correlates with increased hmC and ten-eleven translocation methylcytosine dioxygenase (TET) up-regulation

**Figure 4:**
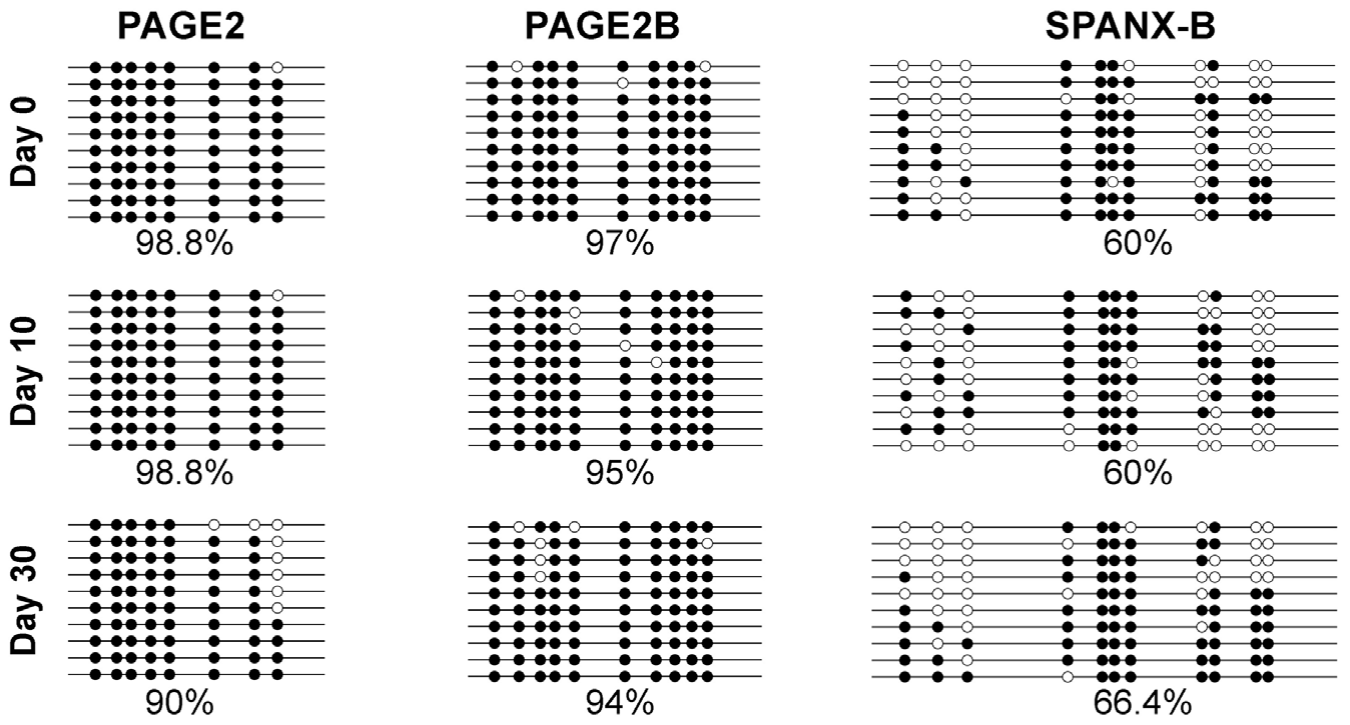
Bisulfide sequencing of *PAGE2, -2B* and *SPANX-B* promoter-proximal regions. Filled and empty circles represent methylated and unmethylated cytosines, respectively. % methylation within each analysed region, based on the 10 clones sequenced is indicated. CpG residues proximal to *PAGE2, -2B* and *SPANX-B* promoters during Caco2 differentiation at days 0, 10 and 30 reveals no statistically significant change (by one-way ANOVA).

**Figure 5:**
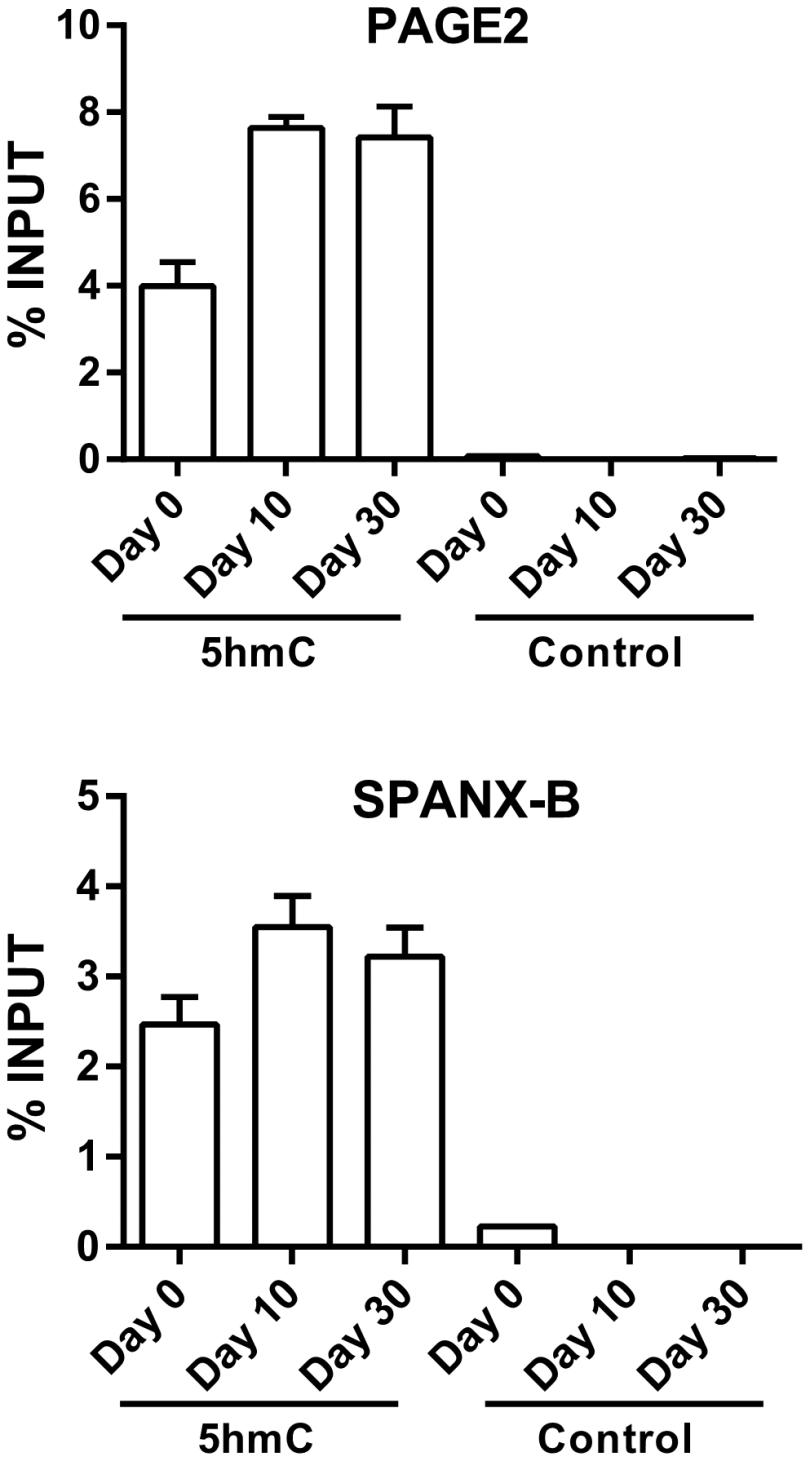
Increased hydroxymethylation of *PAGE2* and *SPANX-B* during Caco-2 spontaneous differentiation. CHIP experiments using an anti-hmC antibody and primers corresponding to +31 to +182 and +68 to +184 bp from the transcription start site of the *PAGE2* and *SPANX-B* genes, respectively. P values (one-way ANOVA) are 0.001 and 0.07 for PAGE2 and SPANX-B, respectively.

**Figure 6:**
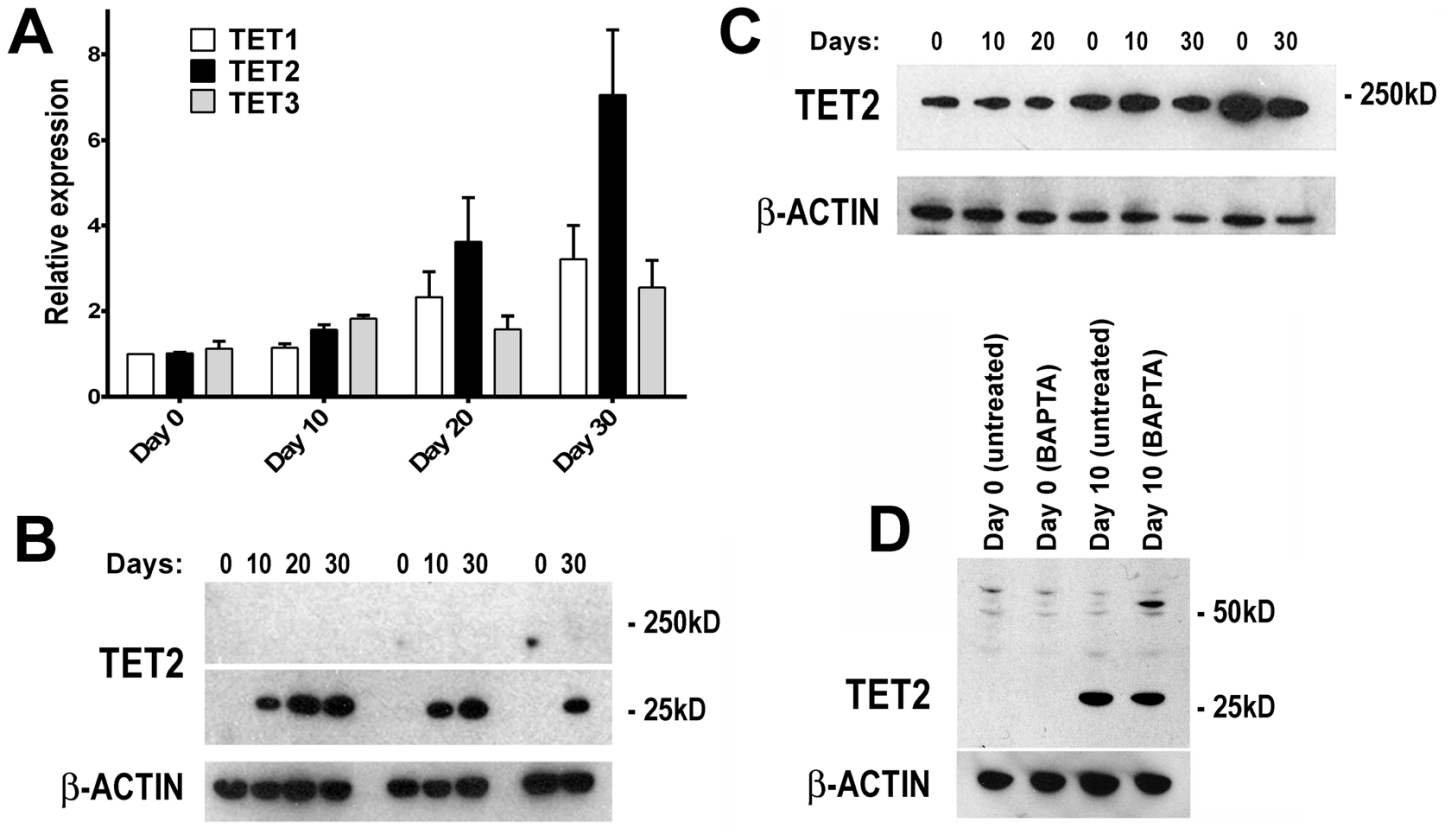
*TET* expression during Caco-2 SD. mRNA of all three *TET* genes increase gradually during Caco-2 SD (**A**). An increase in only a 25 kD version (**B**), but not the full-length TET2 protein (**C**) occurs simultaneously with the increase in mRNA. BAPTA-AM treatment results in a modest decrease in the 25 kD TET2 protein, with the generation of a larger mw version (**D**). P values, as determined by one-way ANOVA, are 0.03, 0.01, and 0.07, for Tet1, Tet2, and Tet3, respectively.

**Figure 7:**
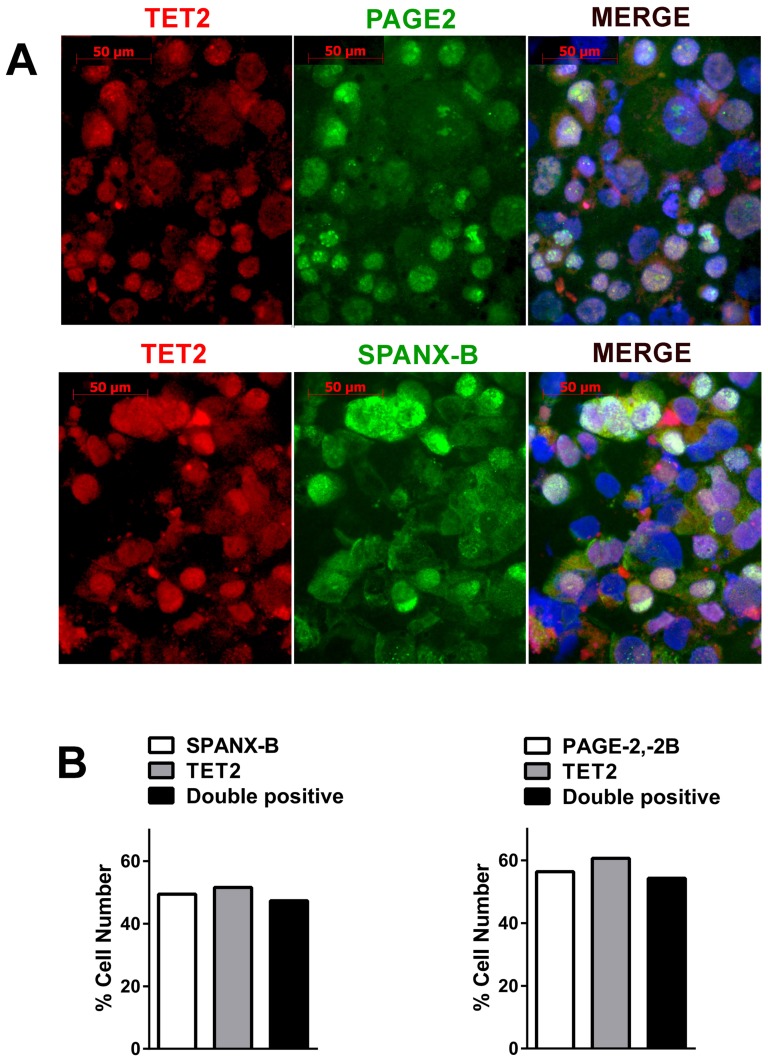
Overlapping TET2 and CT gene expression in differentiating Caco-2 cells. Double immunofluorescence staining for TET2 (Alexafluor 568: red) and SPANX-B or PAGE2 (Alexafluor 488: green) with DAPI counterstaining 20 days post-confluence show overlapping nuclear expression Magnification: 40× (**A**). More than 95% of cells expressing PAGE2 or SPANX-B were also positive for TET2 staining (**B**).

Expression of all CT genes studied thus far including *PAGE2* and *SPANX-B* have been associated with the demethylation of CpG residues within regions proximal to the transcription start site [1], [2], [23]–[26]. In this line, both *PAGE2* and *SPANX-B* can be up-regulated by 5-aza 2′-deoxycytidine treatment (**Figure S8**). However, bisulfite sequencing of promoter-proximal regions of both *PAGE2* and *SPANX-B* revealed no differences at different stages of Caco-2 SD (**Figure 4**). As bisulfite sequencing is unable to distinguish methyl cytosine (mC) from hmC, we asked whether the change in CT gene expression could be related to altered hmC/mC ratios within their promoters. In fact, chromatin immunoprecipitation (ChIP) with a hmC specific antibody revealed an increase in hmC during differentiation in both *PAGE2* and *SPANX-B2* promoters (**Figure 5**). We next asked if the increase in hmC was related to an increase in TET1, -2, and -3 expression as these proteins are responsible for converting mC to hmC [27], [28]. Indeed, the increase in hmC of *PAGE2* and *SPANX-B2* promoters were correlated with an up-regulation of *TET2* mRNA expression, together with modest increases in *TET1* and *3* (**Figure 6A**). Double immunofluorescence staining revealed that the majority of cells expressing PAGE2 or SPANX-B were positive for TET2 staining; indicating these two events occurred in the same cells (**Figure 7**). It is therefore, likely that the increase in TET2 expression causes increased hmC in these genes. Interestingly, only a low molecular weight translation product (∼25 kD) of TET2 was increased in the differentiating cells, when no clear difference in levels of the full-length TET2 protein was observed (**Figure 6B & C**). The peptide used for generating the commercial TET2 antibodies could specifically inhibit recognition of the 25 kD product confirming its identity with TET2 (data not shown). TET2 has recently been shown to undergo proteolytic cleavage by calpain 1 and 2, generating a 25 kD product *in vitro* [29]. To test if a cation-dependent protease is responsible for the generation of the 25 kD protein, we treated differentiating cells with an intracellular Ca^2+^ chelator (BAPTA-AM). This resulted in a modest reduction in the 25 kD TET2 protein with the concomitant generation of a larger molecular weight product (∼50 kD), suggesting that the 25 kD TET2 protein is a Ca^+2^ dependent protease cleavage product with a 50 kD intermediate (**Figure 6D**).

### EZH2 and HP-1 occupancy of *PAGE2* and *SPANX-B* promoter proximal regions decrease during differentiation

**Figure 8:**
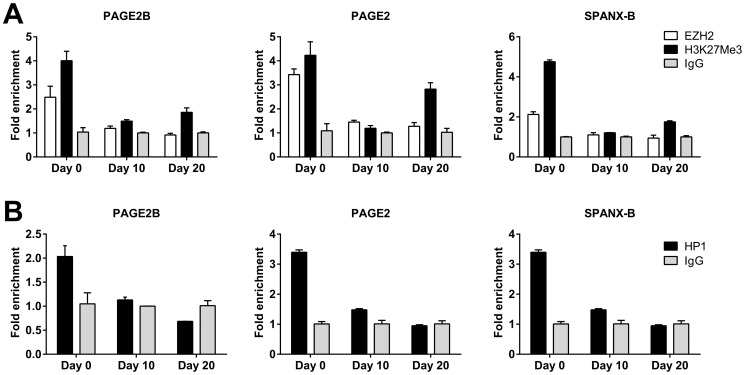
Chromatin modifications within *PAGE2* and *SPANX-B* during Caco-2 differentiation. CHIP analysis of *PAGE2, -2B* and *SPAN-X* transcription-start site-proximal regions reveals decreased EZH2 occupancy and H3K27m3 (**A**), as well as decreased HP-1 binding during differentiation (**B**). P values (one-way ANOVA) calculated for *PAGE2B, PAGE2*, and *SPANX-B*, are <0.001, 0.02, and 0.001 for EZH2; 0.003, <0.001, and <0.0001 for H3K27m3; and 0.001, <0.001, and <0.001, for HP1, respectively.

Hydroxymethylation has been reported to prevail in promoters with dual H3K4 and H3K27 trimethylation that also bind PRC2 proteins [30]. The PRC2 complex protein EZH2 has been implied in the repression of GAGE, another CT gene [31]. We therefore, asked whether increased hmC within CT gene promoters resulted in altered EZH2 binding to the same sites. Indeed, ChIP experiments demonstrated a decrease in EZH2 occupancy, as well as a decrease in H3K27m3 in both *PAGE2* and *SPANX-B* promoters during Caco-2 SD (**Figure 8**). The PRC2 component SUZ12 has been reported to regulate H3K9 methylation and in turn, heterochromatin protein 1 (HP1α) binding. In fact, we observed a simultaneous decrease in HP1 binding to both *PAGE2* and *SPANX-B* promoters during differentiation, that correlated with *PAGE2* and *SPANX-B* upregulation (**Figure 8**). Thus, our data suggest that both PRC2 and HP-1 contribute to maintaining *PAGE2* and *SPANX-B* in a transcriptionally silent state when the cells have a mesenchymal phenotype, and that increased TET2 expression and hmC mediated transcriptional activation are related to PRC2 and HP-1 dissociation from the promoters of these CT genes during differentiation.

### *PAGE2* and *SPANX-B* up-regulation is reversed during EMT

**Figure 9:**
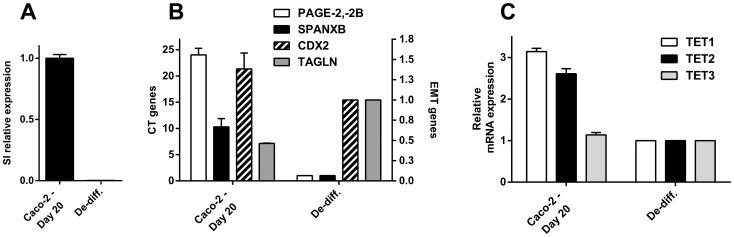
De-differentiation induced EMT and down-regulation of *CT* and *TET* genes. De-differentiation induced by growth under non-confluent conditions indicated by decreased *sucrose isomaltase* (SI) mRNA levels (**A**), leads to down-regulation of *CDX2*, *PAGE2, -2B* and *SPANX-B*, with concomitant up-regulation of *TGLN* (**B**). *TET1* and *-2* mRNAs are also down-regulated during de-differentiation (**C**).

We hypothesized that if the epigenetic alterations underlying CT gene expression happened in parallel to MET, that this process could be reversed if cells entered EMT. To test this hypothesis, differentiated Caco-2 cells were detached and allowed to proliferate for 5 days. This resulted in their rapid de-differentiation as evidenced by down-regulation of SI. De-differentiated cells down-regulated *PAGE2* and *SPANX-B*, as well as *CDX*, as they up-regulated *TAGLN*, in line with ongoing EMT (**Figure 9**). Although transcription of all three *TET* genes decreased during de-differentiation (**Figure 9**), we did not observe a decrease in hmC during this period (data not shown). We, therefore, conclude that the up-regulation of CT gene expression is reversible in this model.

## Discussion

Previous studies revealed that CT gene expression correlated with an epithelial rather than a mesenchymal phenotype, and showed the up-regulation of CT genes during MET [12], [14]. To our knowledge, this is the first report describing alterations in several epigenetic mechanisms within promoters of two CT genes during MET-like differentiation concordant with a dynamic change in gene expression. As bisulfite sequencing of *PAGE2* and *SPANX-B* promoters revealed no change upon differentiation, the increased hmC must strictly involve methylated CpG residues. This is in line with the fact that TET enzymes are responsible for the conversion of 5-methyl cytosine to 5-hydroxymethyl cytosine [27], [28]. Conversion of hmC to mC is a far more complex process and might not happen with similar kinetics [32], [33]. This is likely the reason why we did not observe a change in hmC during the 5 day de-differentiation process of Caco-2 cells despite the decrease observed in global TET levels. Our finding that *PAGE2*, *SPANX-B* and *TET2* induction is reversible is similar to another study in embryonic stem cells where Vitamin C was shown to induce TET2 expression, which in turn, resulted in up-regulation of CT genes. Both events were reversible upon Vitamin C withdrawal [34].

Although hydroxymethylation within gene promoters has been reported to decrease during differentiation of normal cells, a recent study revealed that about 20% of all modified cytosines in most CT genes in human brain, where they are not expressed, consist of hmC [35]. Up-regulation of TET2 expression in cancer has been associated with MET [36], [37]; and therefore, a more differentiated state [30]. Similar to the inverse correlation between EZH2 and CT/TET2 expression we report here, others have shown EZH2 and TET enzymes to repress and induce differentiation of neuronal precursors, respectively [38]. CT genes are up-regulated during the initial stages of development in the human embryo, but decrease as tissues differentiate further [39]. As adult colon tissue does not show PAGE2 or SPANX-B expression (data not shown), had Caco-2 cells the capability of differentiating further, both genes might have been down-regulated completely. On the other hand, the fact that we could not demonstrate up-regulation of *GAGE, MAGE-A3, NY-ESO-1* or *SSX4* expression in this model might be because these genes are expressed at earlier stages of differentiation. We believe this because SPANX-B expression is primarily in post-meiotic cells of the testis (i.e. spermatocytes, spermatids, or sperm), whereas GAGE, MAGE-A3, NY-ESO-1 or SSX expression is primarily in spermatogonia [11].

Our data and that of several others' indicate that cancer cells that express CT genes have more of an epithelial rather than a mesenchymal phenotype. We suggest that CT genes *PAGE2* and *SPANX-B* are induced during a window of differentiation that correlates with up-regulation of epithelial markers of differentiation. The Caco-2 SD model has made it possible to observe the actively changing epigenetic landscape within the promoters of these CT genes. However, as CT gene expression in tumors has closely been related to the methylation state of their promoter, the process that leads to CT gene induction i*n vivo* might ultimately result in “fixing” of the epigenetic state which would in turn result in CpG methylation. Yet, via dynamic MET in tumors [40], it is conceivable that even this might change over the course of the disease.

From a clinical perspective, data from our lab as well as from others reveal that sub-grouping of tumors based on gene expression profiles can clearly identify cells with different chemo-sensitivity profiles [12], [41], [42]. In this line, we predict future studies will reveal distinct drug sensitivity profiles for colorectal cancer subtypes as possibly defined by *PAGE2* and *SPANX-B* expression, for which the Caco-2 SD model could be used.

## Supporting Information

Figure S1 **Post-confluence differentiation of Caco-2** ***in vitro*** **.** Up-regulation of *sucrase-isomaltase* (**A**), and carcinoembryonic antigen (CEA) (**B**) in cells collected at indicated days post confluence (DPC) as determined by quantitative RT-PCR, and Western analysis, respectively. Alkaline phosphatase expression is also upregulated as determined by immunohistochemistry revealing differentiation (**C**). Other measures of differentiation for the cells used in this study have been reported previously (ref. 17). *P<0.001 (ANOVA with Tukey's post hoc test).
(DOCX)

Figure S2 **Up-regulation of CT gene expression during Caco-2 spontaneous differentiation** ***in vitro*** **.** Heat map based on 31 probesets in GSE1614 corresponding to 23 CT genes from 7 families. As compared to proliferating cells, gene expression incrementally increases in at confluence (8^th^ day) and further during post-confluence differentiation (15^th^ day).
(DOCX)

Figure S3 **Western analysis of differentially expressed genes during Caco-2 SD.** A gradual increase in SPANX-B and CDX2 in parallel to a decrease in expression of FN, VIM and TGLN up to day 30 post-confluence. Results from 3 independent differentiation experiments are shown.
(DOCX)

Figure S4 **SPANX-B and Fibronectin expression show limited overlap in differentiating Caco-2 cells.** Immunofluorescent staining of differentiating Caco-2 cells with DAPI counterstaining reveals a gradual increase in nuclear SPANX-B (Alexa Fluor 488: green) with a concomitant decrease in cytoplasmic fibronectin expression (Alexa Fluor 568: red); (20× magnification) (**A**). Less than 10% of cells expressing SPANX-B stained for fibronectin at day 0 (**B**).
(DOCX)

Figure S5 **PAGE2, -2B and Vimentin expression are mutually exclusive in differentiating Caco-2 cells.** Immunofluorescent staining of differentiating Caco-2 cells with DAPI counterstaining reveals a gradual increase in nuclear PAGE2, -2B (Alexa Fluor 488: green) with a concomitant decrease in cytoplasmic vimentin expression (Alexa Fluor 568: red); (20× magnification) (**A**). Less than 10% of cells showed double fluorescence when staining was analyzed quantitatively at day 0. At later time points, none of the cells showed double staining (**B**).
(DOCX)

Figure S6 **PAGE2, -2B and fibronectin expression are mutually exclusive in differentiating Caco-2 cells.** Immunofluorescent staining of differentiating Caco-2 cells with DAPI counterstaining reveals a gradual increase in nuclear PAGE2, -2B (Alexa Fluor 488: green) with a concomitant decrease in cytoplasmic fibronectin expression (Alexa Fluor 568: red); (20× magnification) (**A**). Less than 15% of cells showed double fluorescence when staining was analyzed quantitatively at day 0 (**B**).
(DOCX)

Figure S7 **Nuclear co-localization of CDX2 and PAGE2, -2B in differentiating Caco-2 cells.** Immunofluorescent staining of differentiating Caco-2 cells with DAPI counterstaining reveals overlapping PAGE2. -2B (Alexa Fluor 488: green) and CDX2 (Alexa Fluor 568: red) expression; (20× magnification) (**A**). More than 80% of the cells show double-labeling when analyzed quantitatively (**B**).
(DOCX)

Figure S8 **Induction of PAGE2,-2B and SPANX-B gene expression by 5-aza-2′-deoxycytidine in HCT116 (top) and SK-LC-17 cell lines (bottom).** Relative mRNA expression values at indicated time points compared to day 0, as determined by quantitative RT-PCR are shown.
(DOCX)

Table S1 **PCR primers.**
(DOCX)

Table S2 **Primer locations.**
(DOCX)

Table S3 **Antibodies used for IF staining and western blot analysis.**
(DOCX)
