# Supplementary figures and images for: Epigenetic Mechanisms Underlying the Dynamic Expression of Cancer-Testis Genes, PAGE2, -2B and SPANX-B, during Mesenchymal-to-Epithelial Transition
Source: PLoS One. 2014 Sep 17;9(9):e107905. doi: 10.1371/journal.pone.0107905 (PMC4168264; doi:10.1371/journal.pone.0107905)

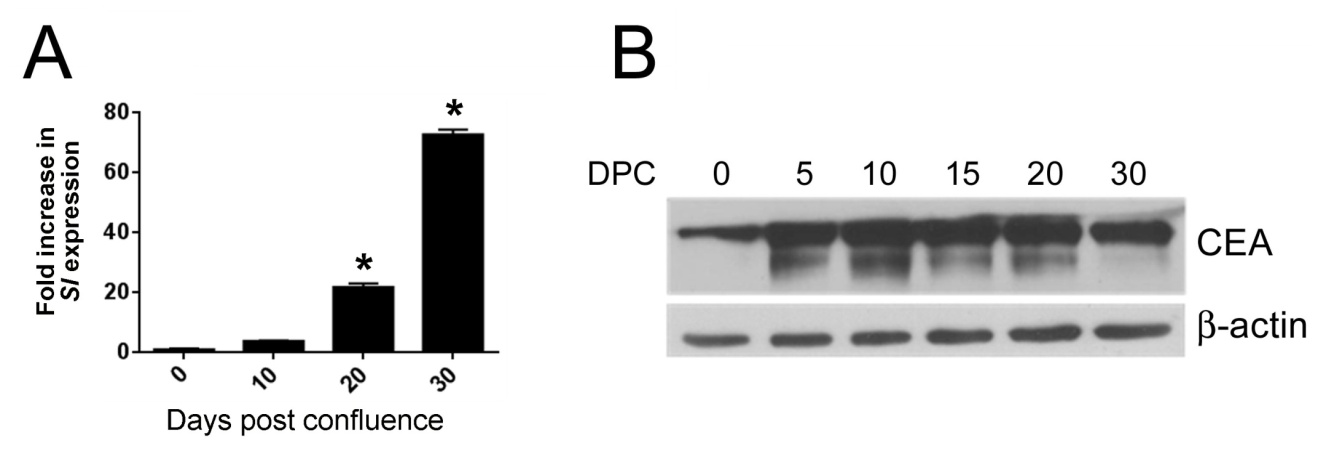


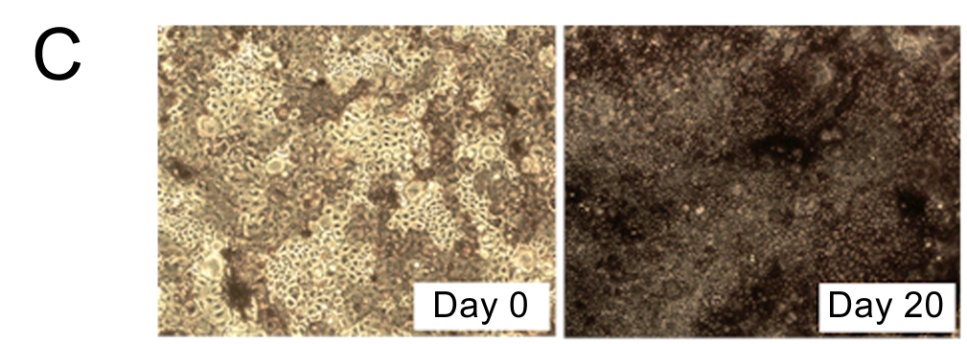

Supplement: Figure S1 — Post-confluence differentiation of Caco-2 in vitro . Up-regulation of sucrase-isomaltase (A), and carcinoembryonic antigen (CEA) (B) in cells collected at indicated days post confluence (DPC) as determined by quantitative RT-PCR, and Western analysis, respectively. Alkaline phosphatase expression is also upregulated as determined by immunohistochemistry revealing differentiation (C). Other measures of differentiation for the cells used in this study have been reported previously (ref. 17). *P<0.001 (ANOVA with Tukey's post hoc test). (DOCX) [file pone.0107905.s001.docx]

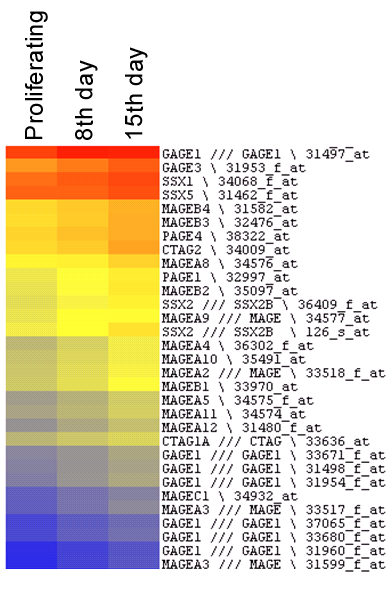


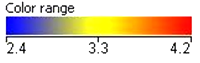

Supplement: Figure S2 — Up-regulation of CT gene expression during Caco-2 spontaneous differentiation in vitro . Heat map based on 31 probesets in GSE1614 corresponding to 23 CT genes from 7 families. As compared to proliferating cells, gene expression incrementally increases in at confluence (8th day) and further during post-confluence differentiation (15th day). (DOCX) [file pone.0107905.s002.docx]

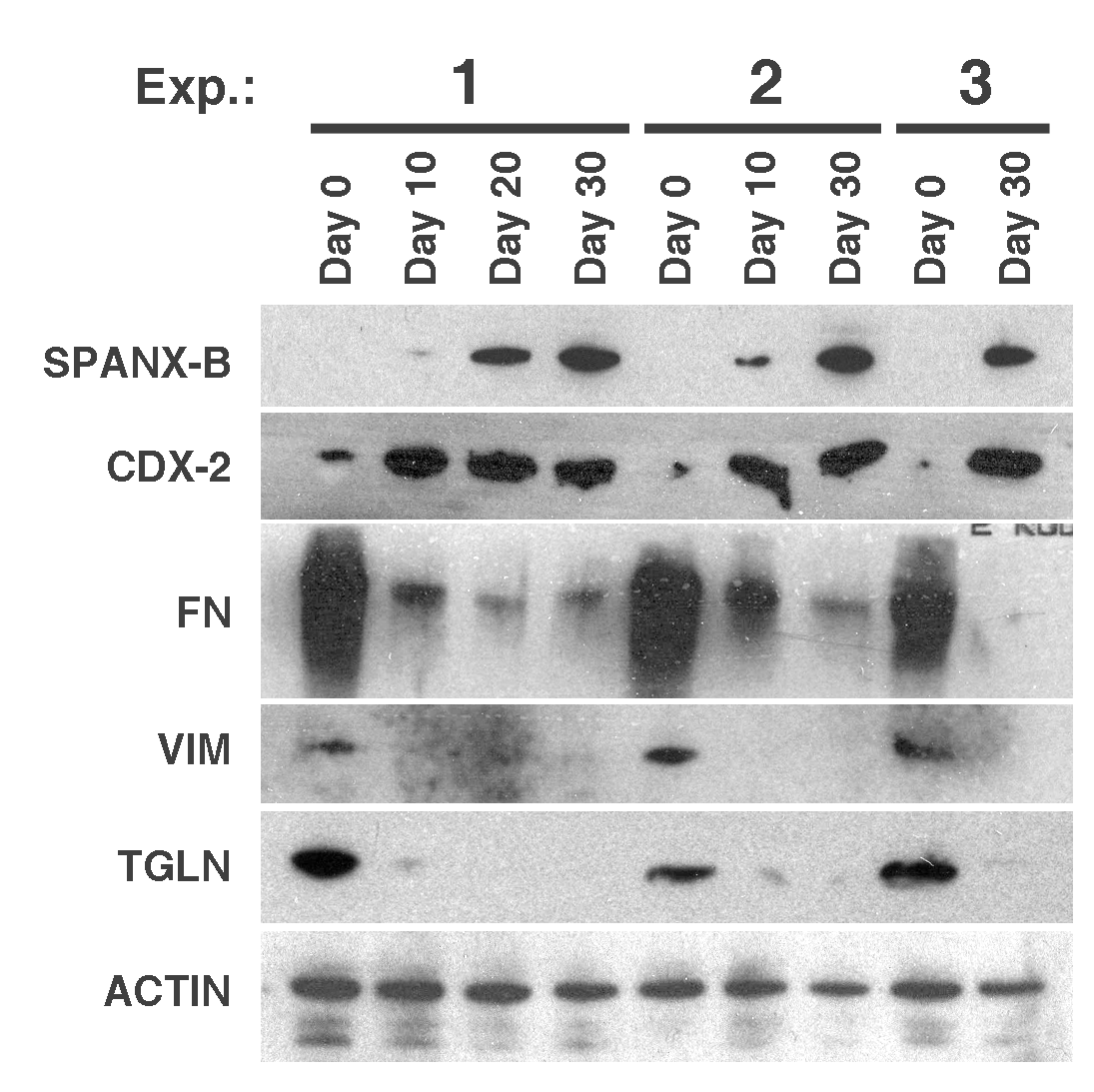

Supplement: Figure S3 — Western analysis of differentially expressed genes during Caco-2 SD. A gradual increase in SPANX-B and CDX2 in parallel to a decrease in expression of FN, VIM and TGLN up to day 30 post-confluence. Results from 3 independent differentiation experiments are shown. (DOCX) [file pone.0107905.s003.docx]

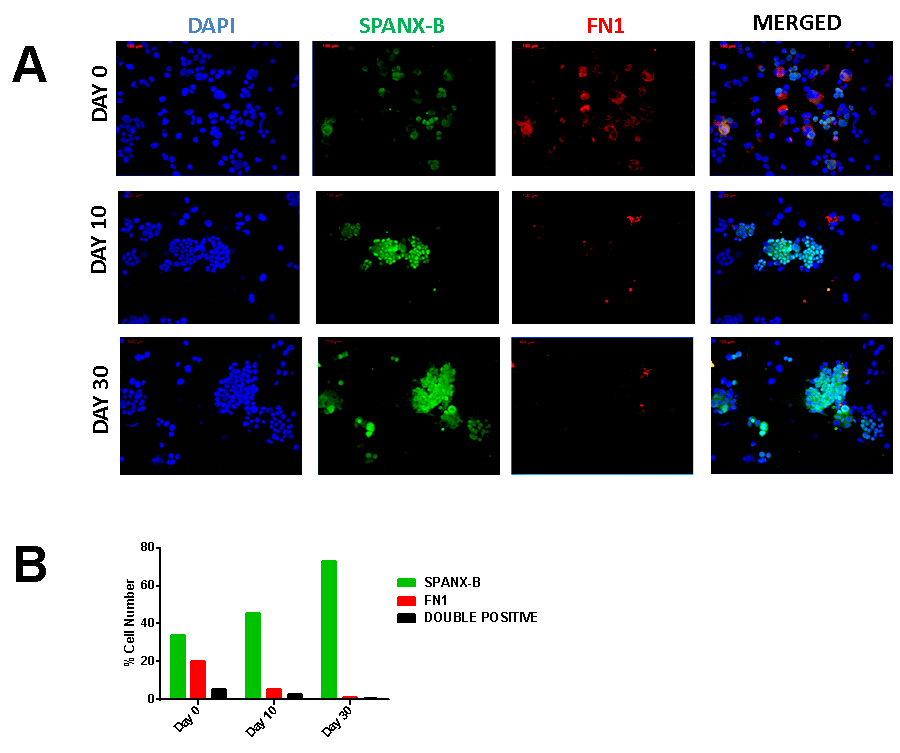

Supplement: Figure S4 — SPANX-B and Fibronectin expression show limited overlap in differentiating Caco-2 cells. Immunofluorescent staining of differentiating Caco-2 cells with DAPI counterstaining reveals a gradual increase in nuclear SPANX-B (Alexa Fluor 488: green) with a concomitant decrease in cytoplasmic fibronectin expression (Alexa Fluor 568: red); (20× magnification) (A). Less than 10% of cells expressing SPANX-B stained for fibronectin at day 0 (B). (DOCX) [file pone.0107905.s004.docx]

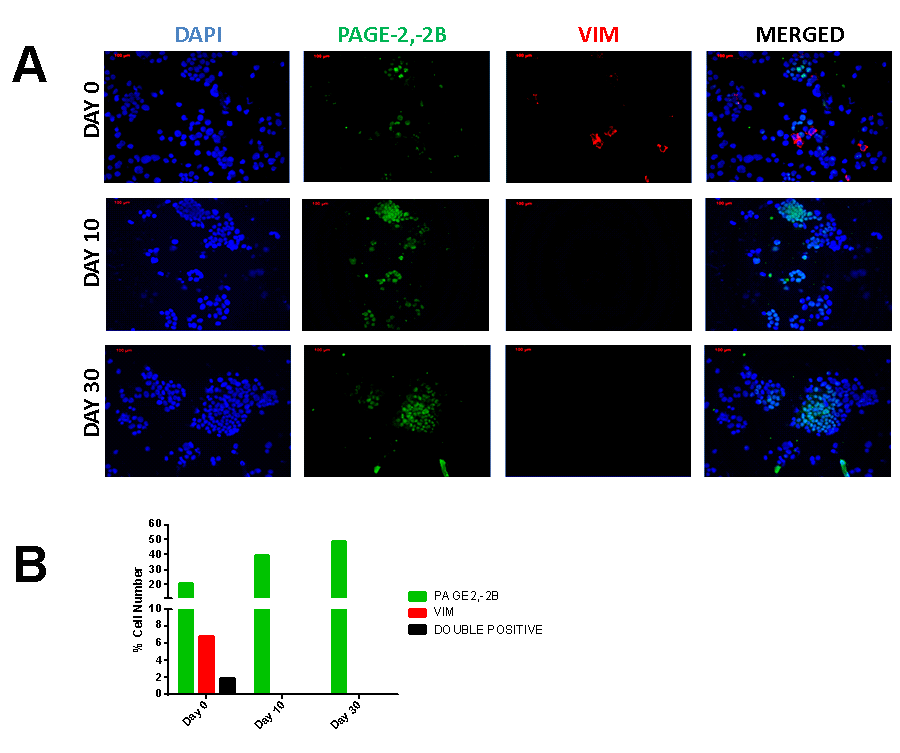

Supplement: Figure S5 — PAGE2, -2B and Vimentin expression are mutually exclusive in differentiating Caco-2 cells. Immunofluorescent staining of differentiating Caco-2 cells with DAPI counterstaining reveals a gradual increase in nuclear PAGE2, -2B (Alexa Fluor 488: green) with a concomitant decrease in cytoplasmic vimentin expression (Alexa Fluor 568: red); (20× magnification) (A). Less than 10% of cells showed double fluorescence when staining was analyzed quantitatively at day 0. At later time points, none of the cells showed double staining (B). (DOCX) [file pone.0107905.s005.docx]

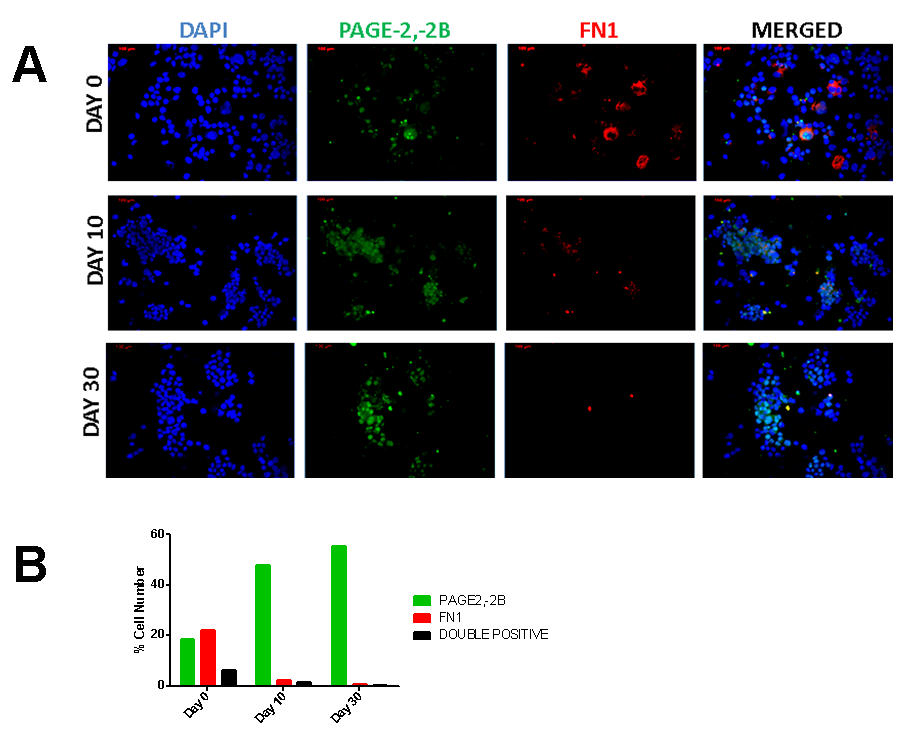

Supplement: Figure S6 — PAGE2, -2B and fibronectin expression are mutually exclusive in differentiating Caco-2 cells. Immunofluorescent staining of differentiating Caco-2 cells with DAPI counterstaining reveals a gradual increase in nuclear PAGE2, -2B (Alexa Fluor 488: green) with a concomitant decrease in cytoplasmic fibronectin expression (Alexa Fluor 568: red); (20× magnification) (A). Less than 15% of cells showed double fluorescence when staining was analyzed quantitatively at day 0 (B). (DOCX) [file pone.0107905.s006.docx]

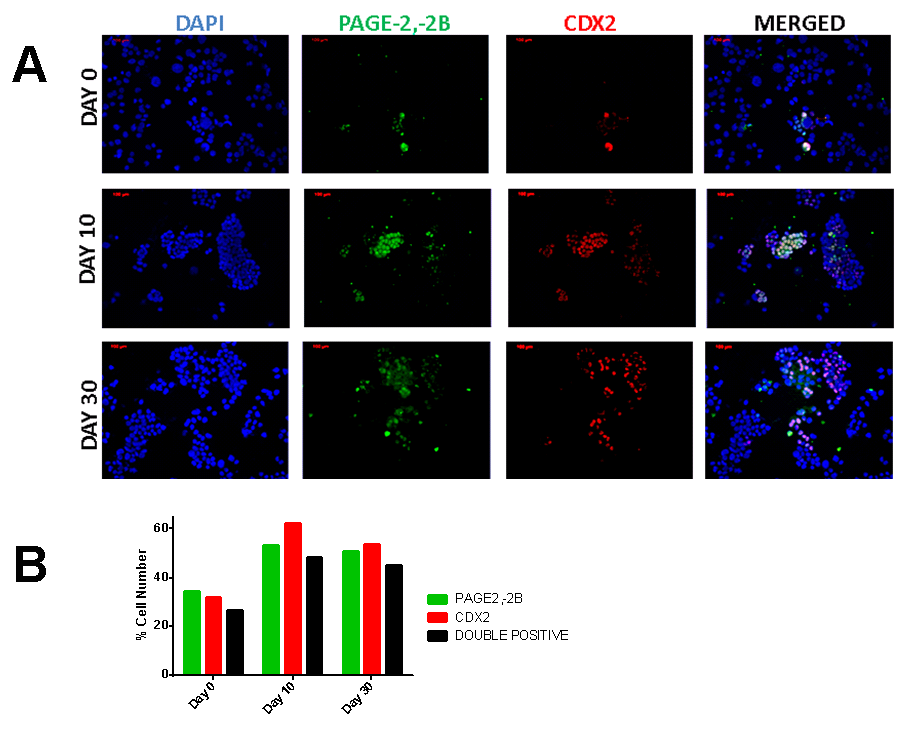

Supplement: Figure S7 — Nuclear co-localization of CDX2 and PAGE2, -2B in differentiating Caco-2 cells. Immunofluorescent staining of differentiating Caco-2 cells with DAPI counterstaining reveals overlapping PAGE2. -2B (Alexa Fluor 488: green) and CDX2 (Alexa Fluor 568: red) expression; (20× magnification) (A). More than 80% of the cells show double-labeling when analyzed quantitatively (B). (DOCX) [file pone.0107905.s007.docx]

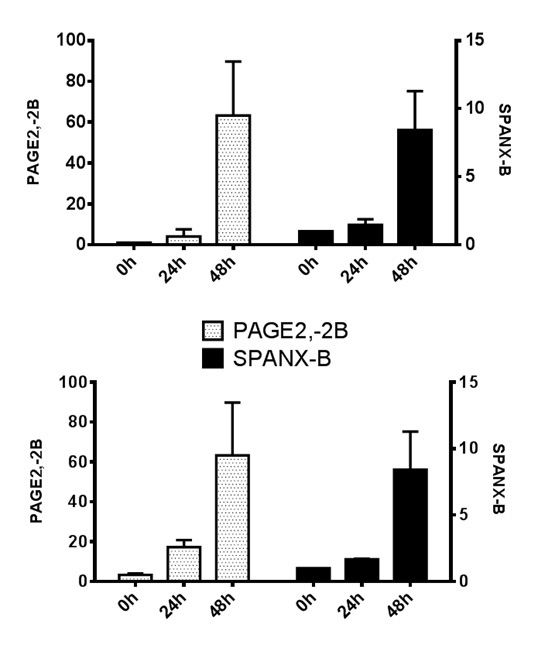

Supplement: Figure S8 — Induction of PAGE2,-2B and SPANX-B gene expression by 5-aza-2′-deoxycytidine in HCT116 (top) and SK-LC-17 cell lines (bottom). Relative mRNA expression values at indicated time points compared to day 0, as determined by quantitative RT-PCR are shown. (DOCX) [file pone.0107905.s008.docx]
