# Supplementary material for: Epigenetic Mechanisms Underlying the Dynamic Expression of Cancer-Testis Genes, PAGE2, -2B and SPANX-B, during Mesenchymal-to-Epithelial Transition
Source: PLoS One. 2014 Sep 17;9(9):e107905. doi: 10.1371/journal.pone.0107905 (PMC4168264; doi:10.1371/journal.pone.0107905)
Supplement: Table S1 — PCR primers. (DOCX) [file pone.0107905.s009.docx]

**Table S1.** **PCR primers**

| **Primer** | **Sequence** | **Product Length** | **Tm** |
| --- | --- | --- | --- |
| ***RT-PCR & Q-RT-PCR*** | | | |
| **GAPDH F** | 5’-TTCTTTTGCGTCGCCAGCCG-3’ | 78 | 61.4 |
| **GAPDH R** | 5’-CGACCAAATCCGTTGACTCCGACC-3’ |  | 66.1 |
| **TAGLN F** | 5’-ACGGCGGCAGCCCTTTAAACC-3’ | 122 | 60.24 |
| **TAGLN R** | 5’-GGCCATGTCTGGGGAAAGAAGGC-3’ |  | 59.74 |
| **FN1 F** | 5’-TGTGATCCCGTCGACCAATGCC-3’ | 131 | 59.23 |
| **FN1 R** | 5’-TGCCACTCCCCAATGCCACG-3’ |  | 59.62 |
| **VIM F** | 5’-CCAAGACACTATTGGCCGCCTGC-3’ | 167 | 60.36 |
| **VIM R** | 5’-GCAGAGAAATCCTGCTCTCCTCGC-3’ |  | 59.42 |
| **CDX2 F** | 5’-CGCTTCTGGGCTGCTGCAAACG-3’ | 262 | 61.65 |
| **CDX2 R** | 5’-TAGCTCGGCTTTCCTCCGGATGG-3’ |  | 60.11 |
| **CLDN4 F** | 5’-ACCTGTCCCCGAGAGAGAGTGC-3' | 157 | 59.4 |
| **CLDN4 R** | 5’-GATTCCAAGCGCTGGGGACGG-3' |  | 60.11 |
| **CDH1 F** | 5’-TGGGCCAGGAAATCACATCCTACA-3' | 91 | 57.57 |
| **CDH1 R** | 5’-TTGGCAGTGTCTCTCCAAATCCGA-3' |  | 57.8 |
| **TET1 F** | 5’-ACCTGCAGCTGTCTTGATCG-3’ | 186 | 60.39 |
| **TET1 R** | 5’-ACACCCATGAGAGCTTTTCCC-3’ |  | 60.27 |
| **TET2 F** | 5’-CGCTGAGTGATGAGAACAGACG-3’ | 187 | 61.29 |
| **TET2 R** | 5’-GCTGAATGTTTGCCAGCCTCG-3’ |  | 62.72 |
| **TET3 F** | 5’-GCATGTACTTCAACGGCTGC-3’ | 187 | 60.18 |
| **TET3 R** | 5’-ATTTCCTCGTTGGTCACCTGG-3’ |  | 60.27 |
| ***BISULPHITE SEQUENCING*** | | | |
| **PAGE2 1A** | 5’-TGGTGGTTTATTTTATAGAGGTAGG-3’ | 342 | 50.1 |
| **PAGE2 1B** | 5’-ACCCTTTTCCCTCAAAAACCA-3’ |  | 51.87 |
| **PAGE2 2A** | 5’-TGTTGGTGTTTATGTTTGTTGTTAT-3’ | 216 | 57.58 |
| **PAGE2 2B** | 5’-ACCAACTAACTCCTCCACACATT-3’ |  | 58.96 |
| **PAGE2B 1A** | 5’-TGGAAGTGAAAGAAAGGGTGGG-3’ | 398 | 54.44 |
| **PAGE2B 1B** | 5’-CAAAACCTATCCAAAACCAACTAACTC-3’ |  | 53.2 |
| **PAGE2B 2A** | 5’-TTGTTGTTGTATTTGTTTGTTGTTA-3’ | 238 | 56.55 |
| **PAGE2B 2B** | 5’-CTATCCAAAACCAACTAACTCCTC-3’ |  | 57.33 |
| **SPANXB 1A** | 5’-CTATCCAAAACCAACTAACTCCTC-3’ | 523 | 53.81 |
| **SPANXB 1B** | 5’-ACCCTCCCTATACATACCCTCC-3’ |  | 53.50 |
| **SPANXB 2A** | 5’-ATTGTAGGAGGGAAATG-3’ | 432 | 52.54 |
| **SPANXB 2B** | 5’-ATTGTAGGAGGGAAATG-3’ |  | 57.39 |
| ***hMEDIP & CHIP Q PCR*** | | | |
| **PAGE2 F** | 5’- GAGCGCTGGTGGTTTACTCC-3’ | 173 | 61.0 |
| **PAGE2 R** | 5’- TCCTTGCAGACCTCTGTGCG-3’ |  | 62.4 |
| **PAGE2B F** | 5’- AGGTTCTCCACAGACGCAGG-3’ | 166 | 61.8 |
| **PAGE2B R** | 5’- TGTGTGTGGACAGAAGGCGG-3’ |  | 62.6 |
| **SPANXB F** | 5’-AACCTACTGTAGACATCGAAGAACC-3’ | 125 | 60.1 |
| **SPANXB R** | 5’-CGTCTTGTTGGCCTCATTGGC-3’ |  | 62.4 |
