# Supplementary material for: Epigenetic Mechanisms Underlying the Dynamic Expression of Cancer-Testis Genes, PAGE2, -2B and SPANX-B, during Mesenchymal-to-Epithelial Transition
Source: PLoS One. 2014 Sep 17;9(9):e107905. doi: 10.1371/journal.pone.0107905 (PMC4168264; doi:10.1371/journal.pone.0107905)
Supplement: Table S2 — Primer locations. (DOCX) [file pone.0107905.s010.docx]

**Table S2: Primer locations**

| **Gene** | **NCBI Reference Sequence**  **(NC_000023.11**  **GRCh38 Primary Assembly)** | **Primer ID** | **Amplified Region (relative to the TSS)** | **Product Size** |
| --- | --- | --- | --- | --- |
| ***BISULPHITE PCR*** | | | | |
| **PAGE2** | 55089064-55092827 | 2A&2B | -188 to +153 | 342 |
| **PAGE2B** | 55075056-55078903 | 2A&2B | -104 to +134 | 238 |
| **SPANXB** | 141002591-141003706 | 2A&2B | -432 to -1 | 432 |
| ***hMEDIP& CHIP Q PCR*** | | | | |
| **PAGE2 (hMEDIP)** | 55089064-55092827 | F&R | +30 to +181 | 152 |
| **PAGE2 (CHIP)** | 55089064-55092827 | F&R | -196 to -24 | 173 |
| **PAGE2B (hMEDIP)** | 55075056-55078903 | F&R | -138 to +75 | 214 |
| **PAGE2B (CHIP)** | 55075056-55078903 | F&R | -176 to -11 | 166 |
| **SPANXB** | 141002591-141003706 | F&R | +68 to +292 | 125 |
