# Supplementary material for: Epigenetic Mechanisms Underlying the Dynamic Expression of Cancer-Testis Genes, PAGE2, -2B and SPANX-B, during Mesenchymal-to-Epithelial Transition
Source: PLoS One. 2014 Sep 17;9(9):e107905. doi: 10.1371/journal.pone.0107905 (PMC4168264; doi:10.1371/journal.pone.0107905)
Supplement: Table S3 — Antibodies used for IF staining and western blot analysis. (DOCX) [file pone.0107905.s011.docx]

**Table S3: Antibodies used for IF staining and western blot analysis**

| Name of the antibody | Supplier | Catalog number |
| --- | --- | --- |
| *PRIMARY* | | |
| Anti-fibronectin antibody | Abcam | ab23750 |
| Anti-vimentin antibody (EPR3776) | Abcam | ab92547 |
| Anti-transgelin (SM22 alpha) antibody | Abcam | ab14106 |
| Anti-CDX2 antibody (AMT28) | Abcam | ab15258 |
| Anti-PAGE-2,-2B antibody (C-13) | Santa Cruz Biotechnology | sc-168892 |
| Anti-SPANX-B antibody (N-13) | Santa Cruz Biotechnology | sc-162267 |
| Anti-TET2 antibody | Abcam | ab-94580 |
| Anti-TET2 antibody | Active motif | 61389 |
| *SECONDARY* | | |
| Alexa Fluor 488 donkey anti-goat IgG (H+L) | Invitrogen | A11055 |
| Alexa Fluor 568 donkey anti-rabbit IgG (H+L) | Invitrogen | A10042 |
| Alexa Fluor 568 donkey anti-mouse IgG (H+L) | Invitrogen | A10037 |
